# Occurrence of Rhabditid Nematodes in the Pet Giant African Land Snails (*Achatina fulica*)

**DOI:** 10.3389/fvets.2019.00088

**Published:** 2019-03-26

**Authors:** Dario d'Ovidio, Jirí Nermut, Chiara Adami, Mario Santoro

**Affiliations:** ^1^Private Veterinary Practitioner, Arzano, Italy; ^2^Institute of Entomology, Biology Centre CAS, České Budějovice, Czechia; ^3^Royal Veterinary College, University of London, Hatfield, United Kingdom; ^4^Dipartimento di Sanitá Animale, Istituto Zooprofilattico Sperimentale del Mezzogiorno, Portici, Italy

**Keywords:** terrestrial gastropod, *Rhabditella axei*, rhabditidae, mollusk parasite, *Achatina fulica*

## Abstract

Gastropods comprise nearly 60,000 species of aquatic as well as terrestrial mollusks, primarily snails and slugs. The giant African land snail (*Achatina fulica*) is one of the most popular pet snails worldwide. This gastropod mollusk is known as the intermediate host of several parasites that cause severe diseases in pets, free-ranging vertebrates, and humans. The aim of this survey was to investigate the occurrence of parasites in giant African land snails kept as pets in southern Italy. Fresh fecal samples were collected from a total of 60 giant African land snails kept in three private collections in Campania region (Italy). In addition, microscopic analysis of mucus and histological examination of biopsy samples from the foot muscle of 30 individual snails were performed. Coprological examination revealed the presence of rhabditid nematodes identified by both morphological and molecular assessment as *Rhabditella axei* in two out of three examined samples, and *Rhabditis terricola, Cruznema* sp., and *Pristionchus entomophagus* in one coprological sample. No parasites were detected in the muscle biopsy samples, or in the mucus. Due to the potential harm of rhabditid nematodes, their presence in giant African land snails potentially in contact with both pets and humans should not be disregarded.

## Introduction

Invertebrates include approximately one million animal species kept both in zoological institutions and in households where they are hand raised as exotic pets (1). Gastropods comprise nearly 60,000 species of aquatic as well as terrestrial mollusks, primarily snails and slugs (1). The giant African land snail (*Achatina fulica*, syn. *Lissachatina fulica*) is native to East Africa, however it is a widespread invasive species in Asia, Oceania, and more recently in Americas, where it has been introduced accidentally or purposefully as a food source and as a pet. Its release in natural ecosystems, agricultural, and urban areas has resulted in ecological, health, and agricultural threats (2, 3). The giant African land snail is an intermediate hosts for several parasites including *Aelurostrongylus abstrusus, Angiostrongylus cantonensis, Angiostrongylus costaricensis, Schistosoma mansoni, Hymenolepis* spp., and *Fasciola hepatica* (4, 5). All the above helminths, with the exception of *A. abstrusus*, are able to cause severe diseases in humans. Specifically, the giant African land snail is the main gastropod responsible for worldwide spread of *A. cantonensis* that causes human eosinophilic meningoencephalitis in Asia and Americas (2, 6). Risk factors for infection in humans, pets and wildlife with those helminth parasites include the ingestion of raw or undercooked infected snails or slugs, or foods contaminated by the slime of infected snails or slugs (4, 5, 7, 8).

Although the African giant land snails are among the most popular snails held as pets, and their worldwide popularity as exotic pets is growing rapidly, only a few surveys have been carried out on the occurrence of their parasites in natural conditions (2, 6, 9–11). The aim of this survey was to investigate the occurrence of parasites in giant African land snails bred as pets in southern Italy.

## Materials and Methods

### General Data

In August 2018, three pools of fresh fecal samples obtained from a total of 60 giant African land snails, kept in three different private collections located in Pozzuoli, Caserta, and Naples (Italy), were investigated for parasites. From each locality we obtained a pool of feces from 20 individual snails each. The snails included in this survey had a median age of 1.6 years ranging from 0.2 to 2 years and had not received any previous anti-parasitic treatment. Snails were fed fresh vegetables and fruits; additionally, a calcium powder supplementation (Calcium, Exo Terra, Hagen Deutschland GmbH & Co. KG, Holm, Germany) was provided twice a week. All animals were bred in Italy, privately owned and kept as pets in four indoor terrariums of 60 × 30 × 45 cm in size, in groups of 10–20 animals (2 groups of 20 snails in Pozzuoli and Caserta and 2 groups of 10 snails each in Naples). An organic peat soil (Organic Coco-Peat Soil, E-Coco Products, Gloucestershire, UK), previously heat-treated (100°C for 30 min) and then free of parasites and insects, was used as substrate.

### Diagnostic Procedures

Twenty grams of fresh feces were initially obtained from each of the four terrarium. When pooled feces analyzed by fresh smear, flotation and Baermann test showed positivity to rhabditid nematodes, two snails from each positive terrarium were housed individually in a sterile plastic box, and their feces were collected, immediately after defecation, in a sterile 50 ml plastic tube. Coprological examination for pooled and individual samples included fresh smear, centrifugal flotation (2 g of feces for each test) using a solution of sugar and formaldehyde (specific gravity 1.27) and Baermann test (10 g of feces for each test). Additionally, the mucus obtained during fresh smear examination was analyzed under a light microscope. Because the larval forms of several helminth species may encyst inside the foot muscle of snails, histological examination of biopsy samples harvested from the anterior and posterior areas of the foot muscle was performed in 30 snails, anesthetized with the technique described by Giannelli et al. (12) and Gilbertson and Wyatt (13). The biopsy samples were fixed in 10% neutral phosphate-buffered formalin, and processed by routine methods into paraffin blocks that were cut into 3 μm thick sections, and stained with hematoxylin and eosin. The study was performed under permission of the practice where it took place and under signed, written informed owner consent.

### Morphological Identification

Temporary mounts were made by heat-killing nematodes on glass slides in a drop of water, after which a glass cover slip was applied. Nematodes were transferred into a drop of tap water on a glass slide and placed on heater (100°C) for 10 s. These specimens were used for morphological identification. An AMPLIVAL light microscope, Carl Zeiss Jena, and a Leitz Diaplan with Nomarski optics were used for observation. Morphological identification of rhabditid nematodes followed Andrassy (14) and Andrassy (15) and it was mostly based on the morphology of pharynx and stoma, and reproductive system, mainly the features of spicules, number and position of papillae or presence and size or shape of bursa for males; and tail shape, position of vulva and morphology of reproductive system for females. The identification of larvae was based on the tail shape.

### Molecular Analysis

DNA was extracted from nematode specimens obtained from feces fixed in 96% ethanol collected from the three localities. Nematodes were washed in double-distilled water (ddH_2_O) overnight, prior to the molecular analysis, for complete removal of residuals of ethanol. Each individual nematode was transferred into a sterile Eppendorf tube (200 μl) with 20 μl of extraction buffer (17.7 μl of ddH_2_O, 2 μl of 10 × concentrated PCR buffer, 0.2 μl of 1% Tween 20 and 0.1 μl of proteinase K). Buffer and nematodes were frozen at −20°C for 20 min, and then immediately incubated at 65°C for 1 h, followed by 10 min at 95°C. The lysates were cooled on ice and then centrifuged (2 min, 9,000 g); 1 μl of supernatant was used for PCR.

A fragment of rDNA containing the internal transcribed spacer regions (ITS1, 5.8S, ITS2) was amplified using primers 18S: 5′-TTG ATT ACG TCC CTG CCC TTT-3′ (forward), and 28S: 5′-TTT CAC TCG CCG TTA CTA AGG-3′ (reverse) (16). A fragment of rDNA containing the gene for 18S rRNA was amplified using primers 22F: 5′- TCC AAG GAA GGC AGC AG GC-3′ (forward), and 1080JR: 5′- TCC TGG TGG TGC CCT TCC GTC AAT TTC-3′ (reverse) (17). The PCR master mix consisted of: ddH_2_O, 7.25μl; 10 × PCR buffer, 1.25 μl; deoxynucleoside triphosphates (dNTPs), 1 μl; 0.75 μl of both forward and reverse primer; polymerase, 0.1 μl; and 1 μl of DNA-extract. The PCR profiles were used as follows: for ITS 1 cycle of 94°C for 7 min followed by 35 cycles of 94°C for 60 s, 50°C for 60 s and 72°C for 60 s, and a final elongation at 72°C for 7 min (14); for 18S 1 cycle of 94°C for 5 min, followed by 35 cycles of 94°C for 60 s, 55°C for 90 s and 72°C for 2 min and a final elongation at 72°C for 10 min. The PCR products were sequenced by GATC Biotech (Germany) and later edited and uploaded to GenBank (https://www.ncbi.nlm.nih.gov/genbank/).

## Results

**Figure 1:**
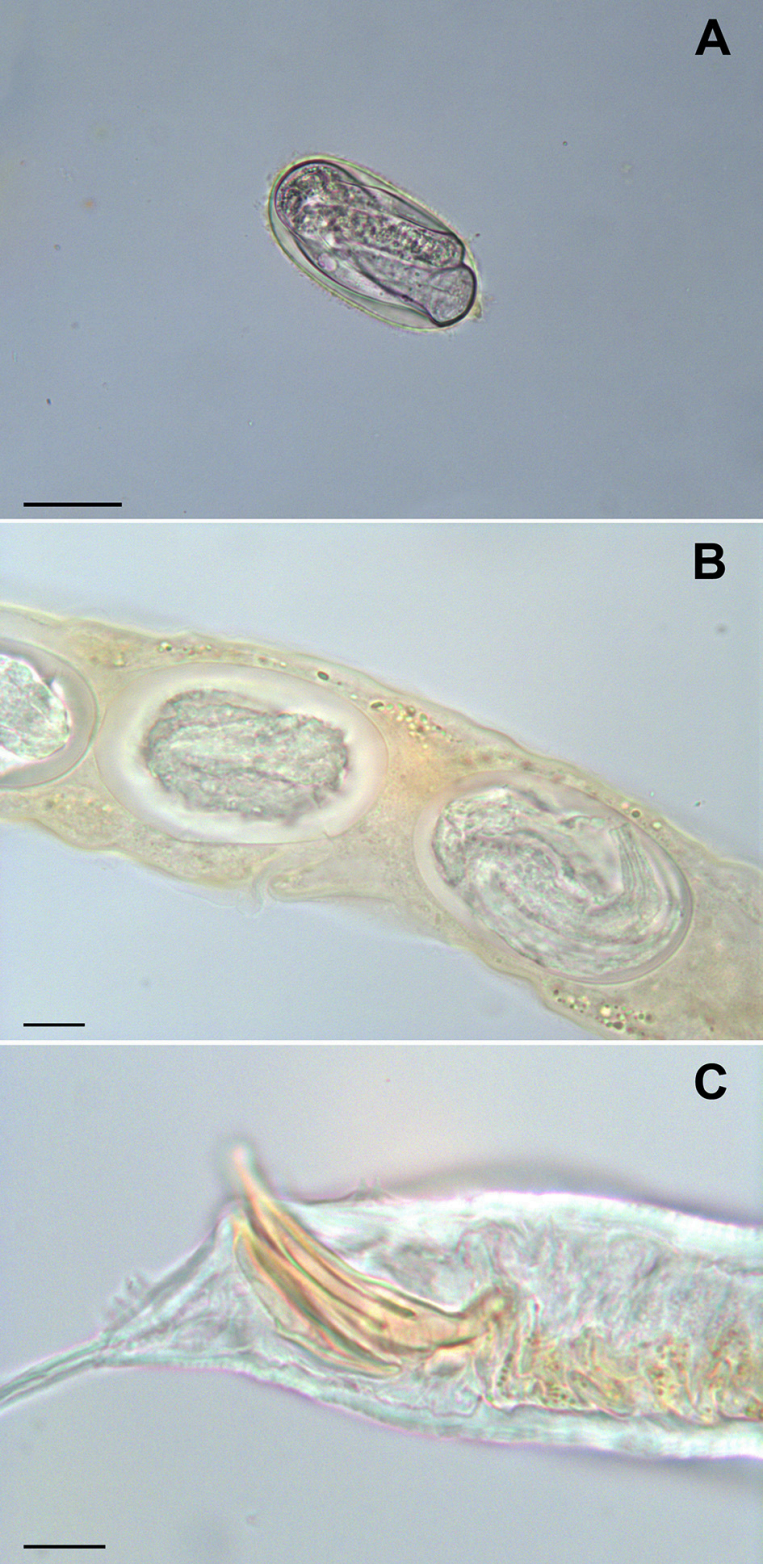
*Rhabditella axei* obtained from the pet giant African land snails (*Achatina fulica*). An egg from coprological examination, bar = 30 μm **(A)** eggs in uterus, bar = 10 μm **(B)** caudal extremity of male, bar = 10 μm **(C)**.

The Baermann test revealed the presence of nematode larvae, whose length ranged from 170 to 336 μm, in all samples. Eggs, larvae and adult rhabditid nematodes were detected by fresh smear and flotation methods in all fecal samples. *Rhabditella axei* (Figure 1) was identified morphologically from two out of three pooled and individual fecal samples from Pozzuoli and Naples. In addition, *Rhabditis terricola, Cruznema* sp., and *Pristionchus entomophagus* were isolated from one pool and individual fecal samples from Caserta. No parasites were detected in the muscle biopsy samples or in the mucus.

Several morphological types of rhabditid nematodes were isolated from snail feces fixed in 96% ethanol. Obtained and edited sequences of ITS and 18S of these nematodes were compared (BLAST) with the material in GenBank and identified as *R. axei* (partial 18S, accession number MK124578, similarity 99%), *Rh. terricola* (partial 18S and partial ITS1, accession number MK156052, similarity 100%), *P. entomophagus* (partial 18S and partial ITS1, accession number MK156050, similarity 99%) and *Cruznema* sp. (partial 18S and partial ITS1, accession number MK156051, similarity 96–100%).

## Discussion

The results of this study indicate that all the examined giant African land snails lay eggs, larvae, and adult rhabditid nematodes in the feces, and may therefore represent a source of infection for other pets and humans. In order to avoid misidentification with *Strongyloides* sp., a nematode species showing close resemblance with rhabditids that has a clear parasitic zoonotic relevance (14, 15, 18), the initial morphological diagnosis of rhabditid nematodes was confirmed through molecular analyses.

Rhabditidae include free-living saprophytic nematodes, widely found in soil and organic debris where they feed mainly on bacteria. Many species of snails may serve as final definitive hosts for rhabditid nematodes (14, 15). However, a number of *Rhabditis* and *Rhabditella* species has been associated with vertebrates including humans (14, 15, 19–27). Although their presence can be the result of environmental contamination, these nematodes may cause diseases in many animals and humans. *Rhabditis elongata, Rh. hominis*, and *Rh. usuii* larvae have been isolated from human feces, urine and vaginal swabs (19–21). Nonetheless, not many cases of symptomatic infections have been reported in humans (21–24). Feng and Li (25) described two human cases of urinary infection by *R. axei* in China, and Ahn et al. (20) reported five human cases of intestinal infection with *Rhabditis* sp. in rural school children of South Korea. Similarly, two cases of human intestinal infection by *R. axei* were described in China (26), whereas another published work (21) reported a case of intestinal infection in a 5-month-old Brazilian child who was presented with fever and watery and bloody diarrhea; coprological examination revealed eggs, larvae and adults of *Rhabditis* sp. Meamar et al. (27) described the occurrence of watery diarrhea in two Iranian patients with AIDS, associated to severe intestinal infection by larvae and adult specimens of *R. axei*. Finally, Teschner et al. (24) recently described a case of outer ear canal infection in a 37 year-old man presented with purulent otorrhea from both ears and acute hearing loss caused by *Rhabditis* sp. In general, *Rhabditis* spp. are considered a common cause of external otitis in cattle living in tropical areas (*e.g*., south America and Africa), particularly in older animals, and have been identified also in chickens, dogs and pigs with incoercible diarrhea (18, 28–30). However, asymptomatic infections have also been described and these nematodes are often considered pseudoparasites (28, 29).

Although free-living nematodes were retrieved in all fecal samples, no specimens were found in the mucus and histological samples. A possible explanation for this finding is that nematodes may locate in different tissues/organs of their hosts, depending on the type of association nematode/mollusk host (15, 31, 32). *Rhabditis* spp. complete its life cycle inside the snail, with no damage to its molluscan host. Previous studies on the free-ranging African snails (*Archachatina* spp. and *Achatina* spp.) revealed that *R. axei* lives in the gastrointestinal tract of its snail host where the entire nematode life cycle is completed (31–33).

Although African giant snails are listed as hosts for *R. axei*, the occurrence of this nematode had been reported only in a few species of giant African snails other than *A. fulica*, namely *Archachatina marginata ovum, Ar. marginata saturalis*, and *Achatina achatina* (31). On the other hand, *P. entomophagus* has a worldwide distribution and has been mainly associated to dung beetles belonging to the superfamily Scarabaeoidea (34, 35), *R. terricola* has been found in salamanders (14, 15) and *Cruznema* spp. in the cricket *Gryllodes laplatae* (Orthoptera) (36). In all cases, transmission of the parasite occurs through the contact of the snails with contaminated moist soil that is rich in decomposing organic matter (31, 37). Although, in the present cases all the animals were kept in terraria with a heat-treated organic soil, we speculate that infection may have occurred before purchase of the animals at the pet shops or breeding facilities.

In conclusion, our results indicate that the pet giant African land snails may serve as reservoir of several rhabditid nematodes. This snail species is among the most commonly ones kept as pets, and therefore often live in close proximity to humans. As a result, the contamination of the domestic environment through their feces is possible. Although parasitic nematodes were not isolated in this study, the giant African land snails should still be considered potential carriers of nematodes able to cause opportunistic diseases in humans. Therefore, we highlight the importance of further epidemiological research on the occurrence of free-living and parasitic nematodes in gastropod snails kept in captivity, and emphasize the need for strict control measures to reduce the risk of opportunistic infection with rhabditid nematodes in pet snail owners.

## Author Contributions

Dd'O and MS conceived and planned the analysis. MS and JN carried out the analysis. CA helped shape the research and edited the manuscript. All authors provided critical feedback and contributed to the final manuscript.

### Conflict of Interest Statement

The authors declare that the research was conducted in the absence of any commercial or financial relationships that could be construed as a potential conflict of interest.
